# Quality by design-based optimization and HP-TLC densitometric standardization of *Theobroma cacao* L. extract as a nutraceutical supplement

**DOI:** 10.3389/fnut.2025.1537963

**Published:** 2025-04-09

**Authors:** Atith Muppayyanamath, Darasaguppe R. Harish, Vinayak Mastiholimath, Priyanka P. Patil, Vishal S. Patil, Harsha V. Hegde, Subarna Roy

**Affiliations:** ^1^ICMR-National Institute of Traditional Medicine, Belagavi, Karnataka, India; ^2^KLE College of Pharmacy Belagavi, KLE Academy of Higher Education and Research (KAHER), Belagavi, Karnataka, India

**Keywords:** quality by design, central composite design, HPTLC, *Theobroma cacao* L, EGCG (−)-epigallocatechin-3-gallate, nutraceuticals

## Abstract

**Background:**

Our previous studies identified the hydroalcoholic extract of defatted *Theobroma cacao* L. bean (CE) as a cancer-preventive and a protective agent against chemotherapeutic-induced toxicities, specifically doxorubicin-induced heart, liver, and kidney toxicities.

**Methods:**

An analytical method for phytochemical standardization was developed, and acute oral toxicity was studied in female Wistar rats following the OECD 423 guidelines. In brief, the CE was extracted using an 80:20 alcohol–water (% v/v) mixture through cold maceration, followed by spray drying to obtain powdered CE. Utilizing a Quality by Design (QbD) approach with Design Expert (DoE) software, we optimized CE tablets via direct compression. The central composite design (CCD) included five center points, with Avicel PH − 101 and croscarmellose sodium (CCS) as factors, and disintegration time, hardness, and % loss due to friability as measurements.

**Results:**

Among the 13 formulations, batch F-9 emerged as the optimized one within the design space, containing 35% Avicel PH − 101 and 5% CCS. The optimized formulation exhibited a disintegration time of 5.2 min, hardness of 4.2 kg/cm^2^, and friability of 0.34%. Importantly, no toxic effects were found at 2,000 mg/kg in the acute oral toxicity study. CE contains vital bioactive polyphenols, including (−)-epigallocatechin-3-gallate (EGCG) and (+)-catechin (CTN). We developed a marker-based HP-TLC densitometric analysis using a mobile phase of 9:9:2 v/v [ethyl acetate: toluene: formic acid], which revealed CTN at Rf 0.49 and EGCG at Rf 0.23. This method was validated according to ICH requirements.

**Conclusion:**

In conclusion, the novel, validated HP-TLC method simultaneously detects EGCG and CTN in the cocoa extract. Tablets formulated by direct compression are safe as nutraceuticals and hold promise as supplements in palliative cancer therapy.

## 1 Introduction

A nutraceutical is defined as a substance derived from food or its components that is used to treat or prevent disease. In 1989, Stephen De-Felice combined the words “nutrition” and “pharmaceutical” to form the word “nutraceutical.” “Nutraceutical is a food, food ingredients, or any dietary supplement that have specific health and medical benefits,” says De-Felice (1, 2). Nutraceuticals have been demonstrated to have antioxidant, anti-inflammatory, anticancer, and lipid-lowering effects (3–5)*. Theobroma cacao* L., widely recognized as cocoa, is not only valued for its culinary uses but also regarded as a medicinal plant; traditionally, the term “cacao” is used to describe the raw components of the *Theobroma cacao* L. fruit, whereas “cocoa” generally refers to its processed forms, and it boasts an array of secondary bio-actives, including flavonoids, terpenes, alkaloids, and polyphenols (6, 7). Among the natural sources of bioactive compounds, cocoa stands out, containing approximately 12–18% polyphenols by dry weight (8, 9). These polyphenols can be categorized into three groups: flavan-3-ols (37%) (including catechins and epicatechins), anthocyanins (4%), and procyanidins (58%) (10). Remarkably, these compounds contribute to the total antioxidant composition in raw, unfermented cocoa beans (11). Beyond its delightful taste, cocoa harbors two key methylxanthines: theobromine (3.7%) and caffeine (0.2%) (12). These compounds were produced through *in vitro* fermentation under controlled conditions to make them fat-free (on a fat-free basis) and to avoid any potential incompatibilities. One study evaluated the protective and anticancer potential of cocoa bean extract (CE) in doxorubicin (DOX)-treated mice with Ehrlich ascites carcinoma (EAC). The results showed that CE selectively targets cancer cells, enhances DOX efficacy, reduces organ damage, and improves antioxidant defenses in mice (13, 14).

Among its various applications, the oral route emerges as the preferred method of drug delivery due to its simplicity, patient compliance, and flexible dose forms (15, 16). Tablets, as unit doses containing active components and excipients, dominate conventional medication administration. However, their rapid breakdown in the digestive tract often leads to elevated plasma drug concentrations. Quality by Design (QbD), guided by ICH Q8-(R2) principles, revolutionizes pharmaceutical development. By emphasizing product and process comprehension, risk management, and continuous improvement, QbD proactively prevents issues rather than reacting to them (17, 18). Key principles include sound scientific risk management, design space establishment, and control strategy implementation. Response surface design (RSD), a powerful QbD tool, offers three variants: central composite design (CCD), Box–Behnken design, and optimal design. CCD, widely employed, features cube, center, and axial points for testing and analysis (19, 20). Its orthogonal blocks facilitate independent variable analysis, minimizing regression coefficient variance. In addition, rotational blocks ensure consistent variance prediction for equidistant points from the center. Statistical models with polynomial or quadratic relationships further explore variables and responses. In this study, we explored methods for developing and optimizing a cocoa nutraceutical supplement in tablet form by leveraging QbD principles and advanced pharmaceutical science (21).

The most advanced form of thin-layer chromatography (TLC) is known as high-performance thin-layer chromatography (HP-TLC) (22). Every stage of the procedure, including exact sample implementation, universally reliable chromatogram development, and software-managed evaluation, uses chromatographic layers with the highest separation efficiency. HP-TLC employs a widely recognized methodology based on scientific principles as well as the utilization of approved quantitative and qualitative analytical methods (23). HP-TLC covers all the quantitative measurement requirements of modern analytical laboratories because of its high resolution and precision (24).

## 2 Methods

### 2.1 Materials

EGCG (99%) and CTN (96%) analytical standards were obtained from Otto Chemicals, Mumbai. Ethanol was sourced from Merck. Petroleum ether (85%) LR grade, methanol, ethyl acetate, toluene, and formic acid HPLC grade solvents were procured from Thermofischer scientific – Navi Mumbai, India. Lactose monohydrate, croscarmellose sodium, and magnesium stearate were supplied by Hi media- Nashik, India Laboratories. Avicel PH − 101 was provided by Molychem- Badalapur, Maharashtra.

### 2.2 Collection and authentication of cocoa pods

Cocoa pods were procured from Kadamba Marketing Souharda Sahakari Niyamita (KMSSN) in Sirsi, Uttara Kannada District of Karnataka, India, and authenticated by the taxonomist at ICMR-NITM Belagavi. The herbarium (voucher number: RMRC−1392) was archived at ICMR-NITM, Belagavi, for future reference.

### 2.3 Extraction and spray drying

Cocoa beans were deshelled, pulverized, and defatted using petroleum ether in a Soxhlet apparatus at 40–60°C. The cycles were repeated until all the fat wax was deposited in the round bottom flask. Subsequently, it was dried at an ambient and dark conditions with 25°C room temperature and pulverized into a coarse powder (25). This powder was sieved through a number 40 sieve to achieve a finer particle size, enhancing extraction efficiency. For extraction, the cold maceration method was obtained using ethanol and water (hydroalcoholic) in a ratio of 80:20 v/v in a conical flask. The mixture was stirred at 100 rpm in an incubator shaker at 37°C for 24 h (26, 27). The liquid extract was filtered, and the filtrate was concentrated using a rota-evaporator (Hei VAP ultimate, Heidolph) at 45°C and 100 rpm. Then, the extract was spray-dried into powder using Spray Mate (Jay Instruments and Systems). The process parameters are shown in Table 1 (28, 29). Physicochemical analysis of extracts, such as loss on drying, total ash, water-soluble ash, acid-insoluble ash, and extractive value, was evaluated, and powder characteristics of the spray-dried CE, such as the angle of repose, Carr’s index, process yield, and hygroscopicity, were also assessed.

**Table 1:** Process parameters used for the spray drying of CE into dry powder.

S. No.	Process parameter	Set value
1.	Inlet temperature (°C)	55
2.	Aspirator speed (rpm)	1,400
3.	Feed-pump speed (rpm)	10
4.	Atomizer pressure (bar)	2
5.	Concentration (%)	20

### 2.4 Compatibility studies

To assess the compatibility of CE with excipients croscarmellose sodium (CCS), Avicel PH − 101, magnesium stearate, and talc to identify any potential interactions, Fourier transform infrared spectroscopy (FT-IR) and differential scanning calorimetry (DSC) were conducted (30). CE and excipients were mixed in a 1:10 ratio (100 mg) scanned immediately and stored at 25 ± 2°C and 60 ± 5% relative humidity for 28 days to observe any degradation. Furthermore, FT-IR and DSC analysis was performed to evaluate the results (31).

### 2.5 Experimental design using DoE

The experiments were structured using Design Expert software version 13.0 (Stat-Ease Inc., Minneapolis, MN, United States). Loss due to friability of not more than (NMT) 1% and disintegration time of NMT 15 min were selected as critical quality attributes (CQAs) for the Quality target product profile (QTPP). In the risk assessment by Quality Risk Management (QRM) approach, increase and reduction in the level of Avicel and CCS revealed a significant impact on the CQAs; thus, Avicel (Binder) and CCS (Super Disintegrant) were found to be independent variables. To develop and optimize nutraceutical tablets, response surface methodology was employed with a face-centered central composite design (CCD). Experimental trials assessed two factors at high and low levels, with 5 center points and axial points (*α* = 1) (32, 33). The independent variables, namely, Avicel PH-101 (X_1_) and croscarmellose sodium (super disintegrant X_2_), were varied at two levels coded as low (−1) and high (+1). The dependent variables, namely, disintegration time (Y_1_) and % loss due to friability (Y_2_), were chosen for the study (Table 2). Thirteen formulations were created using the software (Table 3). The critical values for achieving the desired response from the independent variables were determined using DoE software (34). Response surface plots, including contour and 3D surface plots, were used to illustrate the relationship between dependent and independent variables. Finally, numerical optimization (desirability approach) and graphical optimization (overlay plots) techniques were used to identify the optimized formulation (35–37).

**Table 2:** Selected factor levels and percentages (%w/w) of independent variables combinations as per central composite design (CCD) by Design Expert.

Code	Coded levels		Actual levels (%)	
	X_1_	X_2_	X_1_	X_2_
F-1	−1	−1	25	0.5
F-2	0	0	30	2.75
F-3	0	0	30	2.75
F-4	0	+1	30	0.5
F-5	0	0	30	2.75
F-6	+1	0	35	2.75
F-7	+1	−1	35	0.5
F-8	0	0	30	2.75
F-9	+1	+1	35	5
F-10	−1	+1	25	5
F-11	0	−1	30	0.5
F-12	−1	0	25	2.75
F-13	0	0	30	2.75

**Table 3:** Thirteen compositions of nutraceutical tablets suggested by central composite design (CCD) using Design Expert.

Ingredients	F-1 (mg)	F-2 (mg)	F3 (mg)	F-4 (mg)	F-5 (mg)	F-6 (mg)	F-7 (mg)	F-8 (mg)	F-9 (mg)	F-10 (mg)	F-11 (mg)	F-12 (mg)	F-13 (mg)
CE	150	150	150	150	150	150	150	150	150	150	150	150	150
CCS	0.5	2.75	2.75	5	2.75	2.75	0.5	2.75	5	5	0.5	2.75	2.75
Avicel PH-101	25	30	30	30	30	35	35	30	35	25	30	25	30
Lactose monohydrate	64.5	57.25	57.25	55	57.25	52.25	54.5	57.25	50	60	59.5	62.25	57.25
Magnesium stearate	5	5	5	5	5	5	5	5	5	5	5	5	5
Talc	5	5	5	5	5	5	5	5	5	5	5	5	5
Total weight	250	250	250	250	250	250	250	250	250	250	250	250	250

### 2.6 Formulation of nutraceutical tablets

In this study, the formulation was prepared using the direct compression method. Spray-dried CE and excipients were initially passed through a 60-mesh sieve. All excipients (croscarmellose sodium, Avicel PH-101, lactose monohydrate) were blended with the spray-dried CE in a mortar and pestle using geometric trituration, except for the lubricant. Talc and magnesium stearate (lubricant), which had been previously sieved and accurately weighed, were then added and mixed. Physical properties and pre-compression characterization tests were conducted to evaluate the powder mixture after blending (38). A Rimek 10-station single rotary tablet press with an 8 mm round punch-die set was used to compress the round, biconvex tablets, each weighing an average of 250 mg. Further post-compression evaluation (34), and an acute oral toxicity test was performed according to the guidelines of the Organisation for Economic Cooperation and Development (OECD) (39).

### 2.7 Simultaneous high-performance thin layer chromatography of (−) -epigallocatechin-3-gallalte and (+)-catechin

The analysis used a 20 cm × 10 cm silica gel HP-TLC 60 F254 plate with a fluorescent indicator. At room temperature of 22–25°C, the plate was pre-developed with methanol and dried it using a hand dryer. Using the CAMAG Linomat 5 automated spray applicator, equipped with a 100-μl syringe, the samples and standards (5 μL each) were applied as bands on the plate. The application was performed with an 8-mm band length, a 4-mm band gap, and an 8-mm distance from the y-axis and a 2-mm distance from the x-axis. The application speed was 140 nL/s, with a total of 15 tracks applied. The standard stock solutions of EGCG and CTN were prepared in methanol at a concentration of 1,000 μg/mL. The CE sample stock solution was prepared using a water:methanol (50:50) mixture, resulting in a concentration of 1,000 μg/mL. Ascending development was performed using a mobile phase of 9:9:2 v/v [ethyl acetate: toluene: formic acid] CAMAG twin trough chamber on the spotted plates. The chamber was priorly saturated with mobile phase for 15 min using Whatman filter paper, and the developing distance was 70 mm. After development, the plates were dried using a hand dryer and visualized at R254 and R366 nm using TLC Visualizer II (40). The dried plates were scanned at 254 nm using TLC scanner IV having deuterium and tungsten lamp with Vision CATS software. The process was carried out at a cool temperature of 17–18°C at 40–45% RH, which was maintained throughout the procedure. According to the Q2 International Conference on Harmonisation (ICH) guideline, the established analytical method was validated for linearity, repeatability, limit of detection and limit of quantification (LOD and LOQ), precision, accuracy, and robustness (41, 42).

### 2.8 Statistical optimization

The model’s fit quality was assessed through analysis of variance (ANOVA). This method evaluates the fit by comparing the sums of squares of the residuals and the predicted values. The optimal model was chosen based on various statistical parameters, including the R-squared coefficient, sum of squares (SS), adjusted R-squared, mean squares (MS), *F*-value ratio, degrees of freedom (DF), and *p*-value.

## 3 Results

### 3.1 Physicochemical analysis of spray-dried CE

Macroscopical tests, ash values, extractive values, and moisture content were among the metrics used in the standardization of powdered drugs (Table 4). The spray-dried extract powder was evaluated for the listed parameters, and the results are listed below (Table 5).

**Table 4:** Physicochemical analysis of spray-dried *Theobroma cacao* L. bean extract (CE).

S. No.	Particular	% w/w
1.	Loss on drying	11
2.	Total ash	4.2
3.	Water soluble ash	7.3
4.	Acid insoluble ash	13.6
5.	Extractive value	16.9

**Table 5:** Evaluated results of spray-dried *Theobroma cacao* L. bean extract (CE).

Evaluation parameter	Results
Angle of repose (°)	24.12
Carr’s index	6.2
Process yield (% w/v)	6.17% per 100 mL of extract
Hygroscopicity (% w/w)	11.2

### 3.2 Formulation development

#### 3.2.1 Compatibility studies

**Figure 1:**
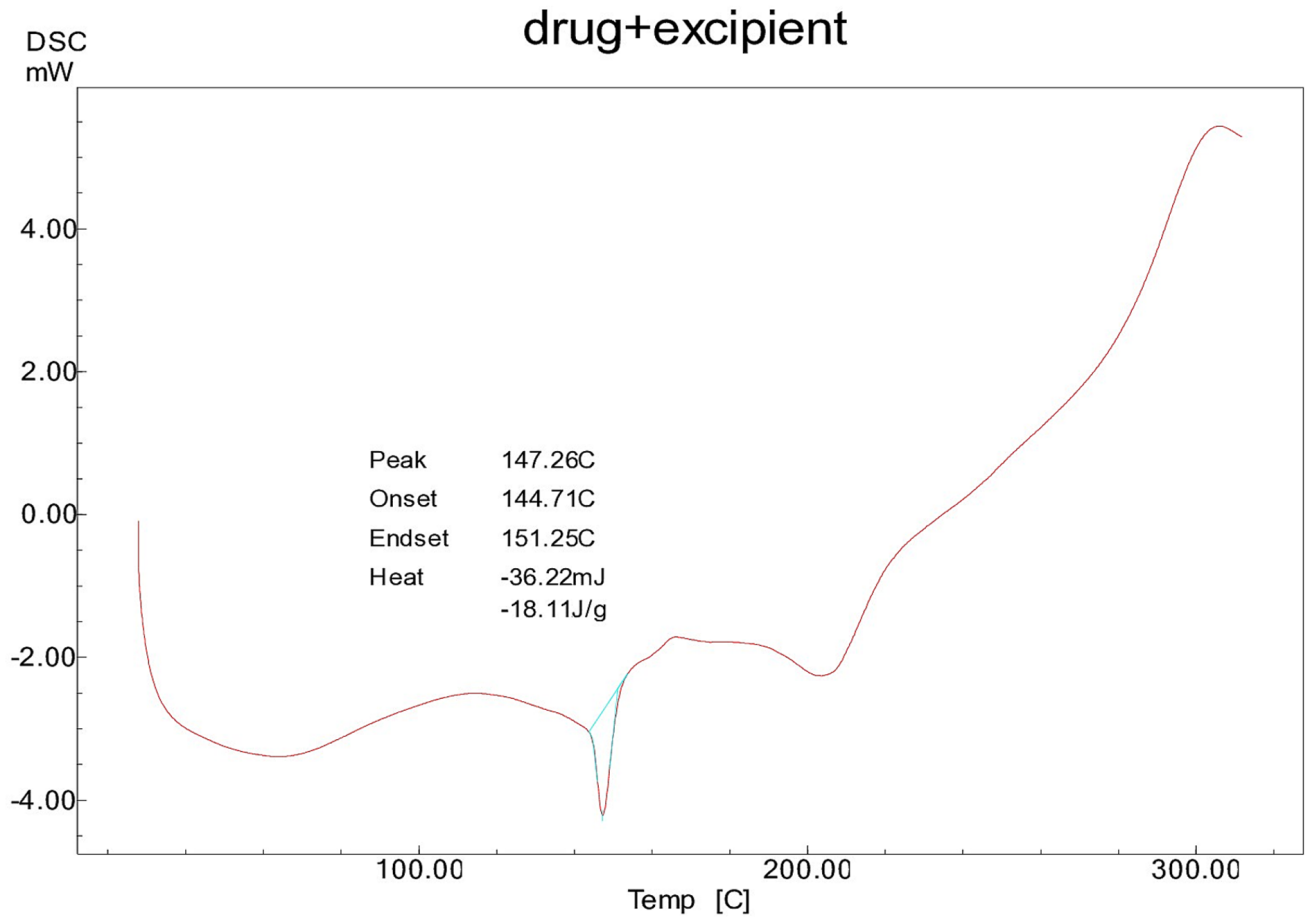
DSC thermogram of physical mixture of spray-dried *Theobroma cacao L.* and excipients in a 1:10 ratio.

**Figure 2:**
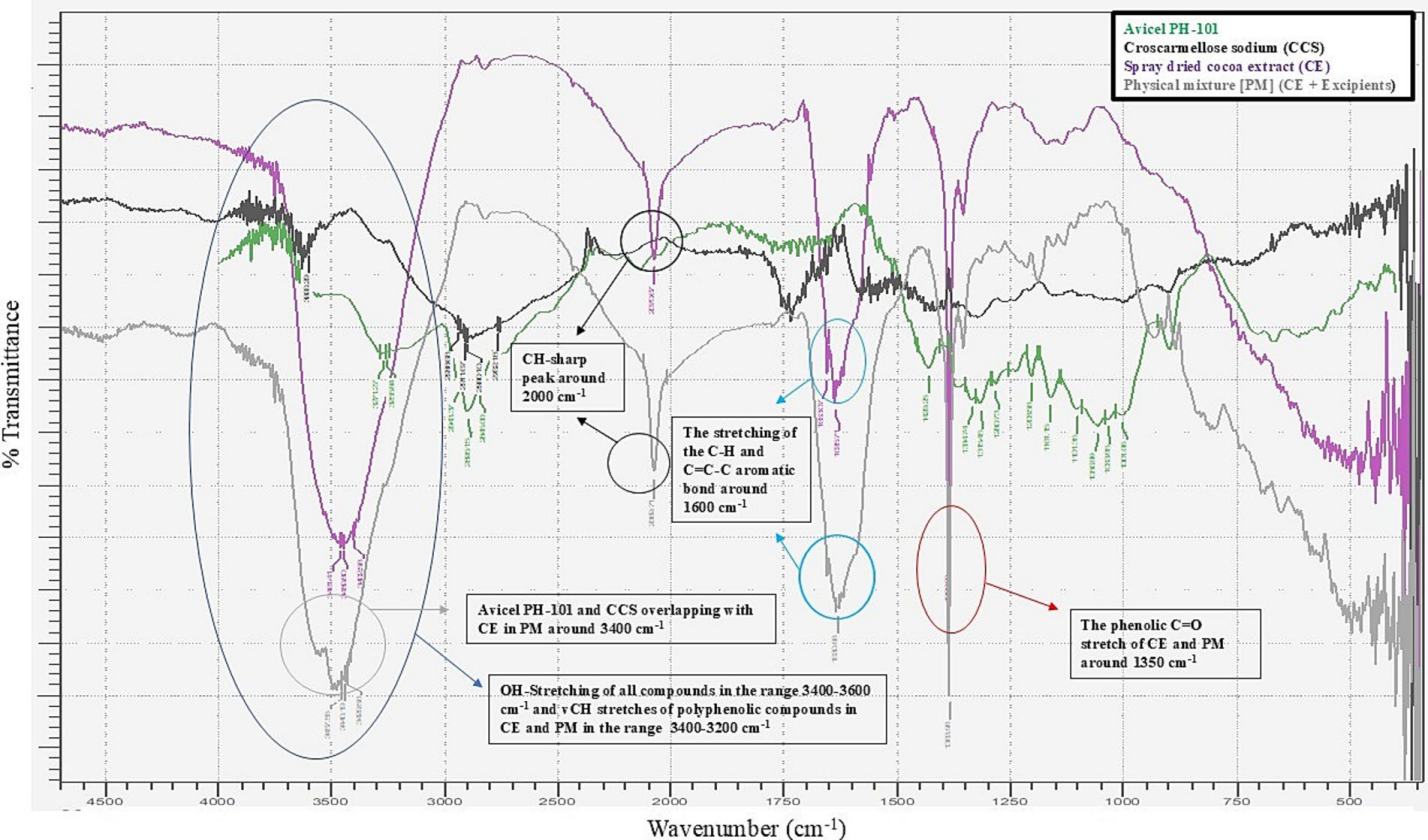
Overlay FT-IR spectra of the CCS, Avicel PH-101, cocoa extract (CE), and physical mixture (extract + excipients in 1:10 ratio).

The DSC analysis of the physical mixture of cocoa extract (CE) with excipients revealed endothermic peaks at approximately 146°C, as shown in Figure 1 The CE exhibited a sharp endothermic peak corresponding to its melting point, which was consistent in the PM and CE samples, indicating that the drug’s thermal properties remained unaltered. However, any significant shift or change in the melting point or appearance of new peaks in the PM or CE could suggest potential interactions between the drug and excipients. In this study, no such significant shifts were observed, suggesting that the components were compatible with each other under the conditions tested. The DSC results confirmed the stability of the formulation and indicated that the excipients did not cause any thermal degradation or chemical interaction with CE. The FT-IR spectra of all compounds show the OH-stretching vibration in the range of 3,400–3,600 cm^−1^, typical of hydroxyl groups. In the case of polyphenolic compounds, such as those present in the CE and PM samples, the vCH stretching is observed in the range of 3,400–3,200 cm^−1^. Notably, Avicel PH-101 and CCS exhibit overlapping peaks with CE in the PM at approximately 3,400 cm^−1^, suggesting a possible interaction or overlapping in functional groups; this suggests that the excipients used in the nutraceutical tablets are compatible with the cocoa extract, as illustrated in Figure 2. In addition, a sharp CH peak appears at approximately 2,000 cm^−1^, which corresponds to specific C-H stretching vibrations. The stretching of the C-H and C=C-C aromatic bonds is evident at approximately 1,600 cm^−1^, indicative of the aromatic nature of the compounds. Finally, the phenolic C=O stretch in both CE and PM is observed at approximately 1,350 cm^−1^, further confirming the presence of phenolic functional groups in the samples.

#### 3.2.2 Experimental design using DoE

Table 3 displays the experimental runs for the developed nutraceutical formulation with independent variables and assessed responses.

**Figure 3:**
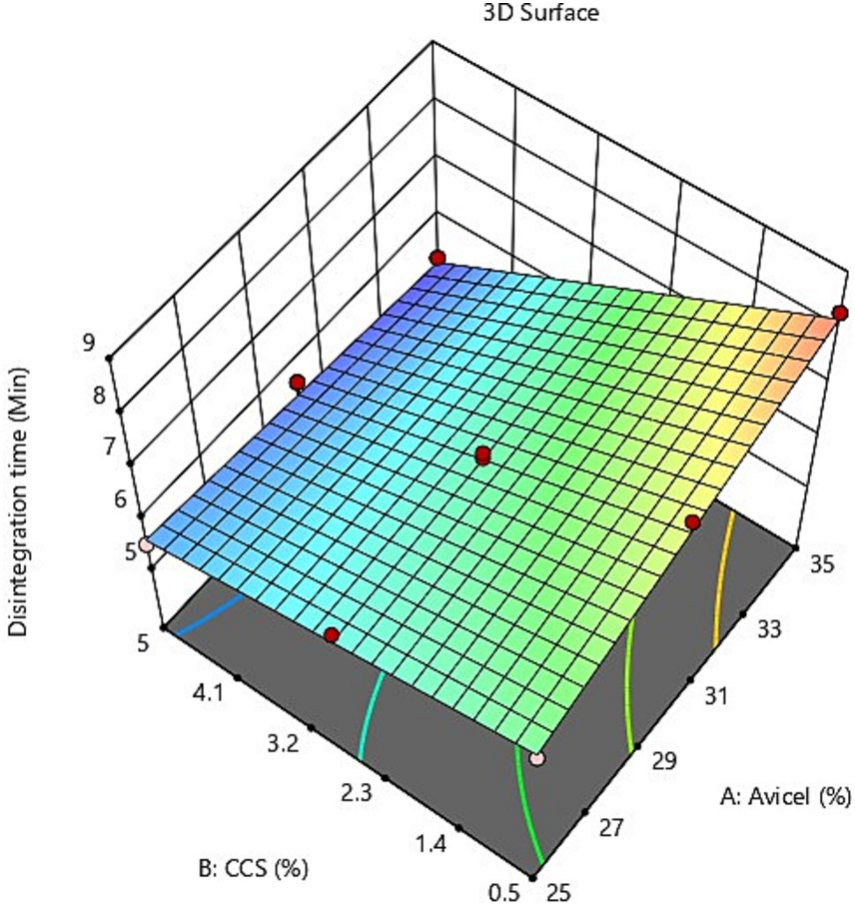
3D surface plot of independent variables on response Y_2_% loss due to friability.

**Figure 4:**
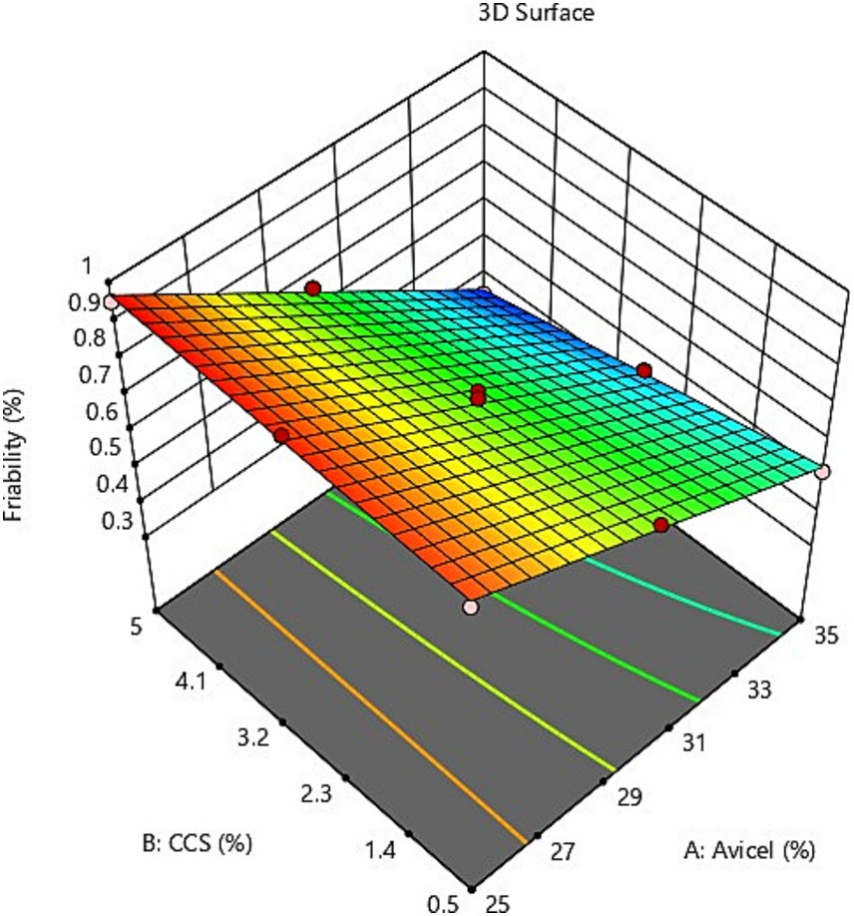
3D surface plot of independent variables on response Y_2_ % loss due to friability.

The evaluation of the quality of fit of the model was executed using the analysis of variance (ANOVA); Y_1_ = +2.1564 + 0.1833111X_1_ + 0.933333X_2_ + 0.046222X_1_X_2_ suggests that the independent variables X_1_ (Avicel PH-101) and X_2_ (croscarmellose sodium) display a positive sign, indicating that they have a synergistic influence on the dependent variable Y_1_ (disintegration time). While for the other dependent variable Y_2_ (% Loss due to friability) = +1.84406 – 0.036889X_1_ + 0.1311111X_2_ + 0.004889X_1_X_2,_ X_1_ is exhibiting a negative sign for its antagonistic influence but variable X_2_ is discovered to have a synergistic effect on the response as evidenced by its positive coefficient, according to response surface plot (Figure 3), and the value of response Y_2_ falls as the quantity of variable X_1_ rises, i.e., as the concentration of Avicel rises, the % loss due to friability lowers. Friability is unaffected by the croscarmellose sodium content. It can be seen from the graphical demonstration (response surface plots in Figures 3, 4), that as the concentration of Avicel and CSS increases, the disintegration time decreases.

#### 3.2.3 Optimization of nutraceutical tablet by central composite design

**Figure 5:**
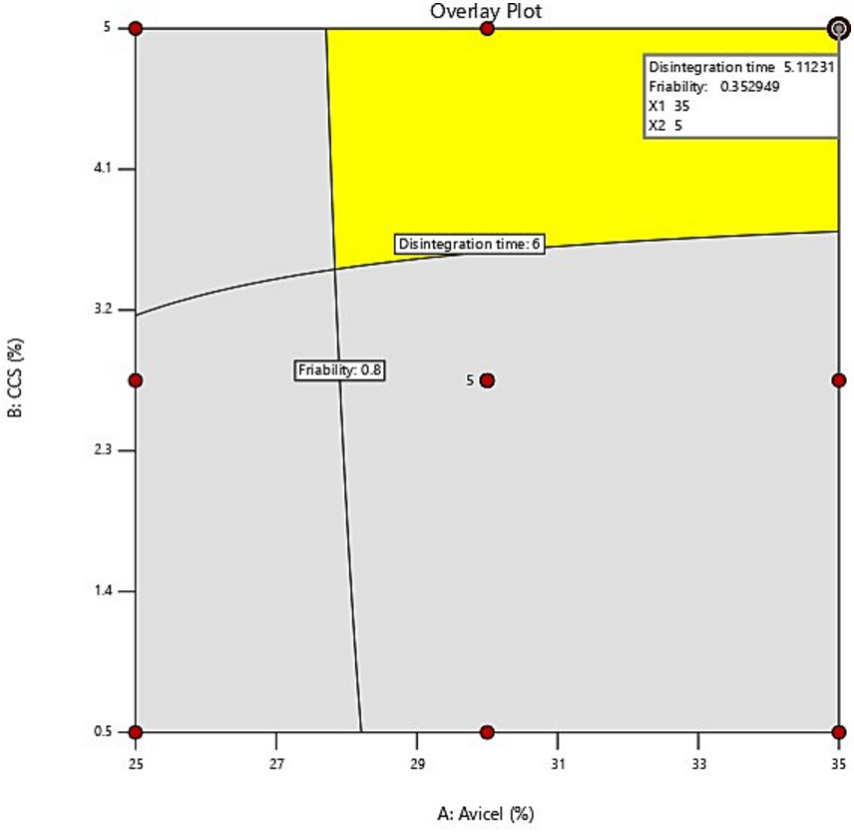
Overlay plot showing design space.

It was discovered that the CCS and Avicel PH-101 levels substantially affected the disintegration time responses and % loss due to friability. The dependent variables selected based on the targeted product quality requirements were disintegration time not to exceed 15 min and loss due to friability not to exceed 1%. The successful operating range region was shown by a darkened zone with yellow in the design space (overlay plot) in Figure 5. From the design space yellow region, X_1_ and X_2_ concentrations already depicted in the design table which is F-9 fall within the range of effective operating conditions. As a result, formulation F9 (CCS-5% and Avicel PH-101-35%) satisfies the requirements of QTPP and CQA for the formulation of nutraceutical tablets. F9 was selected as the optimized formulation, redeveloped, and evaluated for its responses. The expected results as per the Design Expert prediction were a disintegration time of 5.1 min and a loss due to friability of 0.35%. The actual test results for the optimized formula showed a disintegration time of 5.2 min and a loss due to friability of 0.34% (Figure 5).

#### 3.2.4 Pre-compression evaluation (micromeritics study and flowability study)

Pre-compression evaluation involves assessing powder properties such as flowability, compressibility, and density to ensure optimal tablet formulation Data are expressed as mean ± SD (*n* = 3). In this experiment, all 13 formulations indicated good flow properties as Carr’s index was within the range of 9.06 ± 0.416–18.9 ± 0.556%, Hausner’s ratio 1.144 ± 0.020–1.63 ± 0.020, and angle of repose 30.3 ± 0.23–37.3 ± 0.53° (Table 6).

**Table 6:** Pre-compression characterization of powder blend.

Formulation batch	Bulk density (g/mL)	Tapped density (g/mL)	Carr’s index (%)	Hausner’s ratio	Angle of repose (°)
F-1	0.422 ± 0.02	0.522 ± 0.024	14.19 ± 0.361	1.174 ± 0.020	32.2 ± 0.41
F-2	0.467 ± 0.02	0.576 ± 0.020	18.9 ± 0.5567	1.34 ± 0.0450	34.5 ± 0.23
F-3	0.455 ± 0.02	0.545 ± 0.003	9.06 ± 0.416	1.656 ± 0.041	36.3 ± 0.53
F-4	0.445 ± 0.01	0.555 ± 0.007	13.23 ± 0.416	1.553 ± 0.047	36.2 ± 0.55
F-5	0.431 ± 0.02	0.566 ± 0.024	16.59 ± 0.361	1.634 ± 0.020	36.4 ± 0.41
F-6	0.457 ± 0.02	0.575 ± 0.020	10.9 ± 0.5567	1.34 ± 0.0450	36.5 ± 0.23
F-7	0.469 ± 0.02	0.512 ± 0.003	11.76 ± 0.416	1.556 ± 0.041	31.3 ± 0.53
F-8	0.450 ± 0.01	0.596 ± 0.007	15.23 ± 0.416	1.553 ± 0.047	34.2 ± 0.55
F-9	0.424 ± 0.01	0.5240.003	12.12 ± 0.361	1.24 ± 0.0450	30.3 ± 0.23
F-10	0.433 ± 0.02	0.534 ± 0.024	13.59 ± 0.361	1.144 ± 0.020	36.2 ± 0.41
F-11	0.458 ± 0.02	0.533 ± 0.020	18.9 ± 0.5567	1.24 ± 0.0450	36.5 ± 0.23
F-12	0.468 ± 0.02	0.517 ± 0.003	9.76 ± 0.416	1.156 ± 0.041	37.3 ± 0.53
F-13	0.449 ± 0.01	0.523 ± 0.007	16.23 ± 0.416	1.153 ± 0.047	36.2 ± 0.55

#### 3.2.5 Post-compression evaluation

Using the direct compression method, formulation batches F1 through F13 were formulated by changing the amounts of the binder Avicel PH-101 and the super disintegrant croscarmellose sodium. The tablets were compressed, and then, their various physical characteristics were assessed. Data are expressed as mean ± SD (*n* = 3). For physical appearance and color, all formulation batches were assessed. All of the tablets had a uniform appearance that was round and biconvex, brown in color, smooth, and emitted a distinctive odor. The results of the post-compression studies are as follows: thickness of 4.10 ± 0.08 to 4.20 ± 0.07 mm, hardness or crushing strength of 4.1 ± 0.09 to 4.7 ± 0.05 kg/cm^2^, average weight of 252 ± 1.4 to 275 ± 2.3 mg, loss due to friability of 0.35–0.91%, and disintegration time of 5.2 ± 0.02 to 8.3 ± 0.05 min. All physical properties of the formulated tablets of 13 different formulations exhibited acceptable compliance with the compendial specification (Table 7).

**Table 7:** Comprehensive evaluation of the post-compression characteristics of the formulated tablet: weight variation, hardness, friability, and disintegration.

Formulation batch	Thickness (mm)	Weight variation (%)	Hardness (Kg/cm^2^)	Friability (%)	Disintegration time (min)
F-1	4.20 ± 0.07	260 ± 1.4	4.2 ± 0.052	0.91 ± 0.02	6.52 ± 0.01
F-2	4.15 ± 0.06	275 ± 2.3	4.5 ± 0.076	0.65 ± 0.01	6.11 ± 0.08
F-3	4.20 ± 0.08	255 ± 1.5	4.7 ± 0.054	0.71 ± 0.03	6.21 ± 0.09
F-4	4.12 ± 0.08	254 ± 2.6	4.1 ± 0.097	0.67 ± 0.04	5.06 ± 0.04
F-5	4.14 ± 0.07	252 ± 1.5	4.2 ± 0.052	0.71 ± 0.02	6.02 ± 0.07
F-6	4.16 ± 0.08	258 ± 2.3	4.5 ± 0.076	0.45 ± 0.04	6.45 ± 0.06
F-7	4.17 ± 0.06	253 ± 1.5	4.7 ± 0.054	0.52 ± 0.03	8.03 ± 0.05
F-8	4.18 ± 0.07	264 ± 2.6	4.1 ± 0.097	0.73 ± 0.01	6.04 ± 0.03
F-9	4.10 ± 0.08	252 ± 1.4	4.7 ± 0.054	0.34 ± 0.02	5.02 ± 0.02
F-10	4.20 ± 0.09	262 ± 2.3	4.2 ± 0.052	0.95 ± 0.01	5.05 ± 0.04
F-11	4.17 ± 0.04	263 ± 1.5	4.5 ± 0.076	0.74 ± 0.01	7.06 ± 0.05
F-12	4.14 ± 0.05	254 ± 2.6	4.2 ± 0.054	0.96 ± 0.03	6.25 ± 0.09
F-13	4.16 ± 0.02	252 ± 1.4	4.1 ± 0.097	0.69 ± 0.02	6.05 ± 0.06

### 3.3 Acute oral toxicity studies

The approval is obtained from the Intuitional Animal Ethical Committee of ICMR-National Institute of Traditional Medicine (IAEC/ICMR-NITM BGM/Mar3/1), Belagavi, India. Following OECD test guideline 423, a 14-day acute oral toxicity study was conducted on female Wistar rats. Initially, three rats were orally administered 2,000 mg/kg of body weight (equivalent to the CE dosage in nutraceutical tablets). The animals were closely monitored for toxic symptoms such as swelling, discoloration, or lesions on the body, changes in fur condition, color, or any signs of skin irritation, changes in activity levels, aggression, lethargy, or excitability, and mortality for up to 24 h. Subsequently, animals were observed daily for the following 14 days. Body weight was determined each day. Throughout the 14-day observation period, no symptoms of toxicity or mortality were observed, and the animals maintained their regular body weight (39).

### 3.4 Simultaneous HP-TLC method

**Figure 6:**
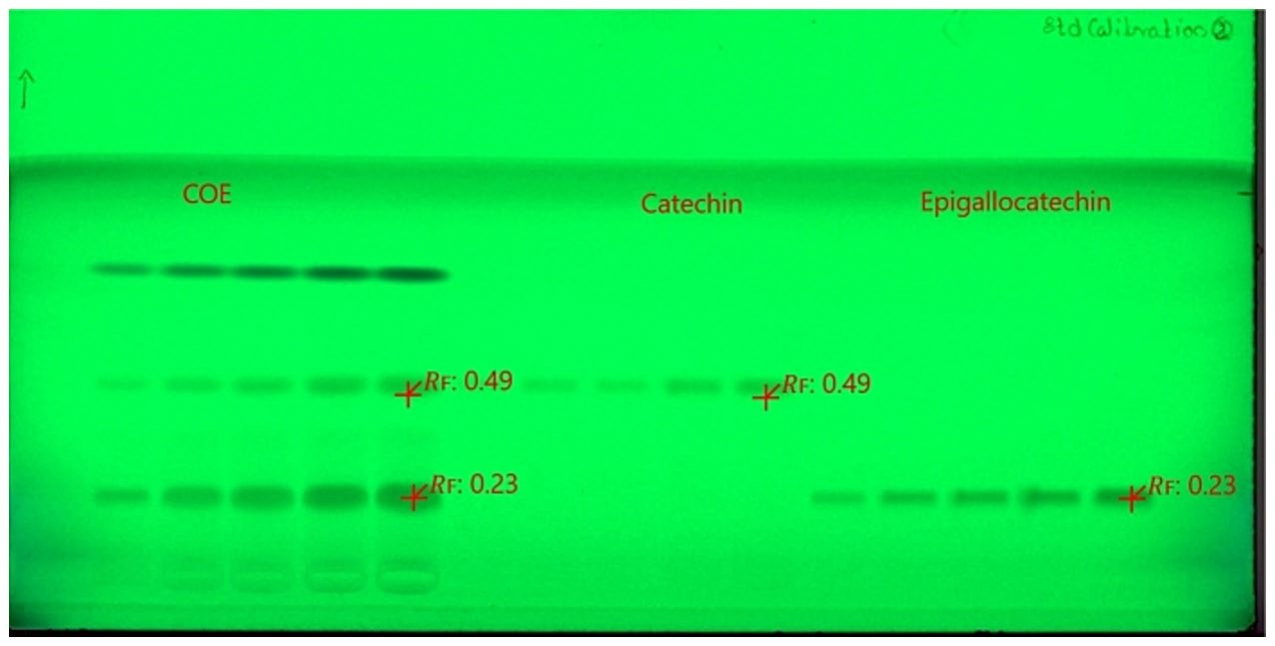
Images of the developed HP-TLC plates for CTN and EGCG.

**Figure 7:**
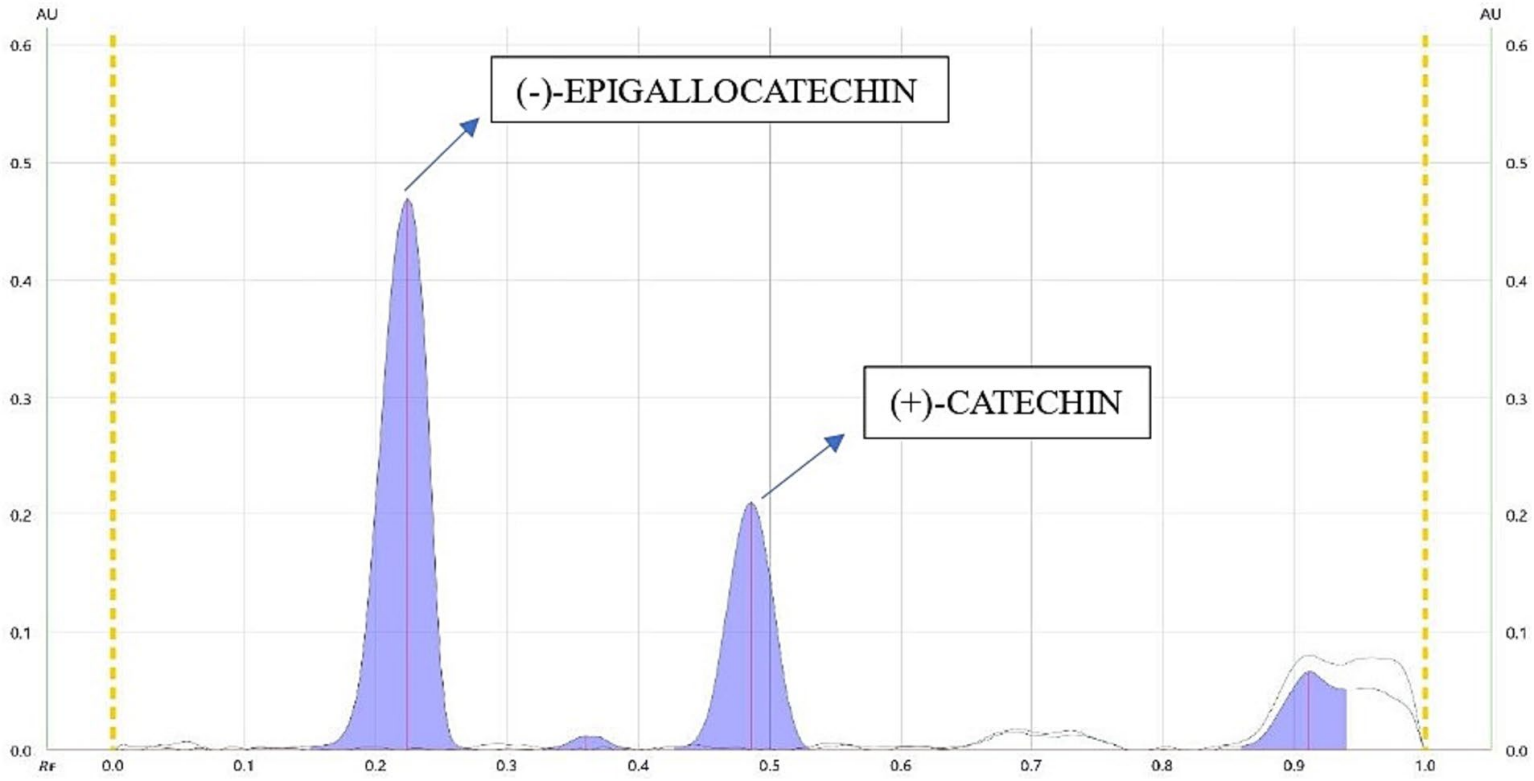
Chromatogram of CTN and EGCG.

**Figure 8:**
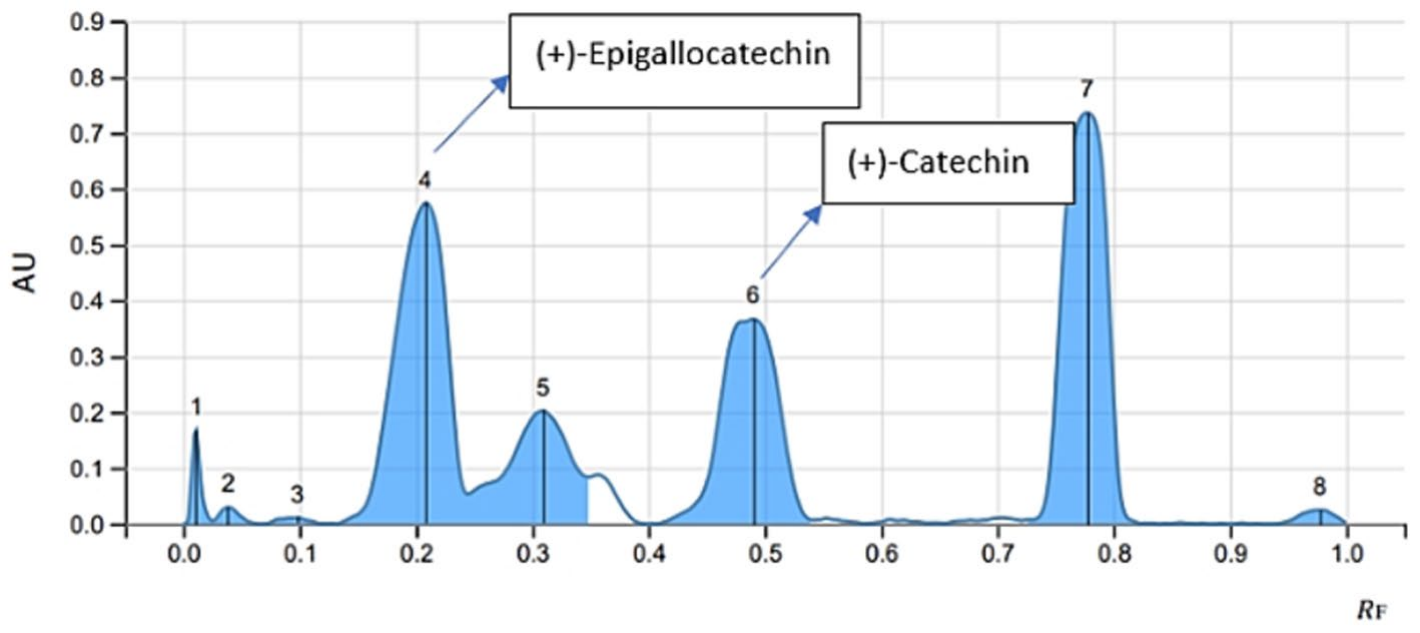
HP-TLC chromatogram depicting CTN and EGCG in CE.

**Figure 9:**
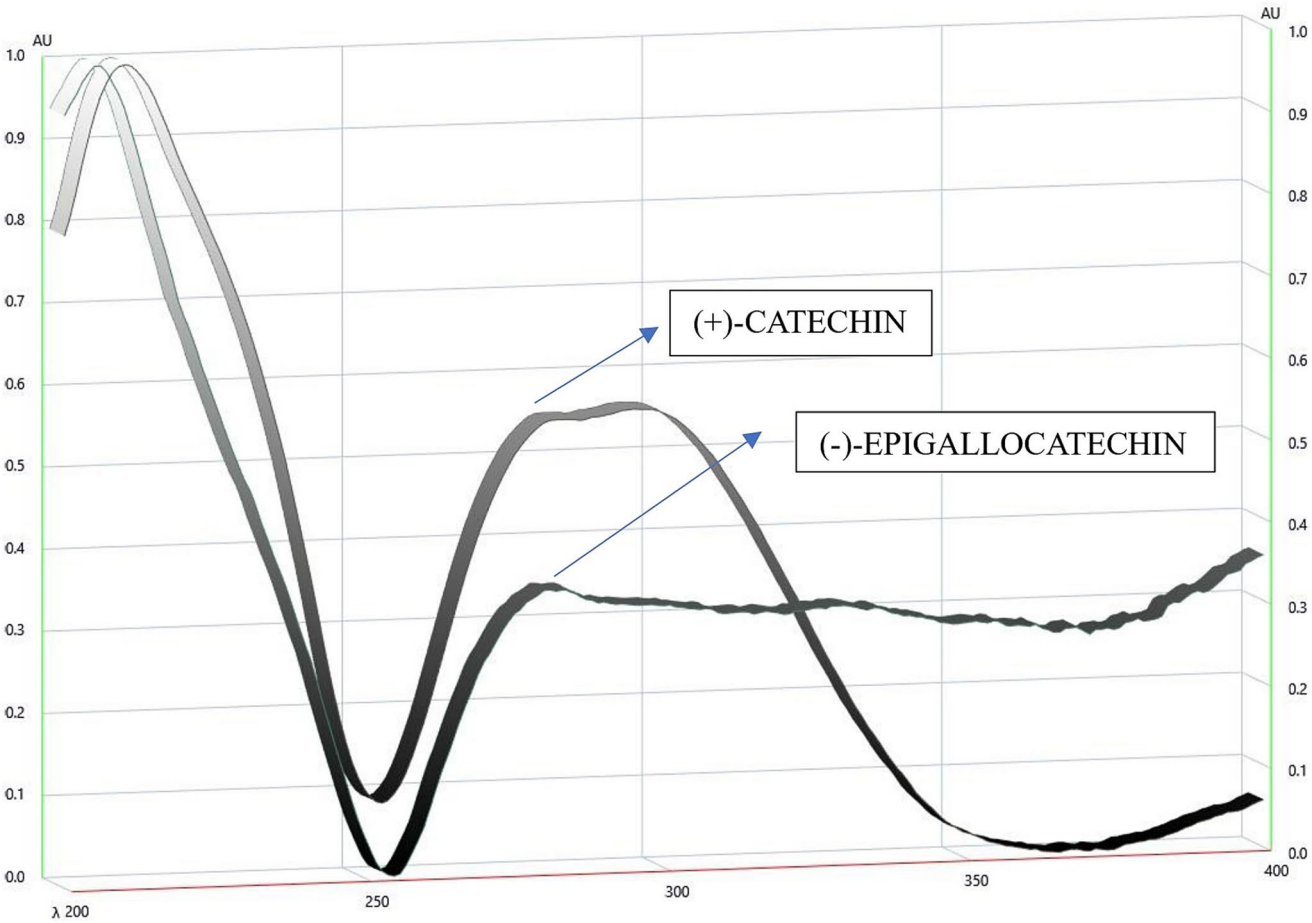
UV-visible spectra of (+)-Catechin and (−)-Epigallocatechin by CAMAGA TLC Scanner 3.

The mobile phase ethyl acetate/toluene/formic acid (9:9:2) produced good resolution of CTN and EGCG. The sample and standards migrated in the mobile phase on pre-activated silica gel plates up to 70 mm, resulting in good band separation (Figure 6). After drying the plates, densitometric analysis was performed at 254 nm. The retention factor value (Rf) is 0.45 (CTN) and 0.29 (EGCG), respectively (Supplementary Figures S1, S2) (Figures 7, 8). The contents of (+)-catechin and (−)-epigallocatechin quantified using TLC densitometric methods were found to be 22.43 μg (CTN) and 18.20 μg (EGCG), respectively. Figure 9 shows the spectrum of both marker compounds, and Table 8 provides a summary of the proposed HP-TLC method’s validation parameters, which were determined to be within the ICH guidelines’ standard limits.

**Table 8:** Evaluation of the developed HP-TLC method: results of studies on validation and analytical performance.

S. No.	Validation parameters	CTN	EGCG
1.	Linearity (ng/mL)	5–25	5–25
	Correlation coefficient	0.999	0.998
2.	LOD (ng/spot)	1.98	3.02
	LOQ (ng/spot)	6	8.94
3.	Precision/Repeatability	0.102	1.762
	Intra-day (%RSD)	0.85	0.3
	Inter-day (%RSD)	0.856	0.30
4.	Accuracy (% Recovery)	102	98.42
5.	Robustness	Within 2%	Within 2%

## 4 Discussion

The present study is based on the findings that the hydroalcoholic extract of *Theobroma cacao L*. beans (CE) exhibits anti-proliferative activity. Furthermore, co-administration of this extract with cancer chemotherapeutic agents, such as doxorubicin, has been shown to reduce the toxicity typically induced by these drugs. In a pioneering study, scientists explored the protective and therapeutic benefits of CE in mice treated with doxorubicin for EAC. The results were significant; CE significantly mitigated doxorubicin-induced organ damage and oxidative stress, showcasing its cardioprotective, hepatoprotective, and nephroprotective properties. When given either on its own or in conjunction with doxorubicin, CE improved vital biomarkers for the heart, liver, and kidneys, while also repairing tissue damage in these organs. Moreover, CE amplified the anticancer efficacy of doxorubicin, evidently slowing the progression of cancer, reducing ascitic fluid accumulation, and extending the median survival time of the mice. These compelling findings reveal CE’s potential as a nutraceutical or complementary therapy in cancer treatment, offering dual benefits of antioxidant and anticancer effects while minimizing the adverse effects of doxorubicin (43, 44). CE was investigated for its potential as a supplement with analgesic properties in combination with paracetamol. The study, conducted on a neuropathic pain model using mice, demonstrated that the combination significantly reduced both pain levels and inflammation. A strong correlation was observed between pain reduction and decreased TNF-*α* levels, highlighting the anti-inflammatory benefits of the treatment. These findings suggest that combining cacao bean extract with paracetamol could be an effective approach for managing neuropathic pain and inflammation (45).

Nutraceuticals, derived from food sources, offer health benefits beyond basic nutrition. Available as tablets, capsules, or liquids, they reduce dependency on pharmaceuticals and minimize side effects (46). Cocoa, rich in antioxidants, enhances tablet taste and stability and provides health benefits, making it valuable in nutraceutical formulations for palliative therapy (47). Research on cocoa as a formulation is limited; however, a few studies have explored its use as an excipient, as mentioned earlier. Orally disintegrating tablets (ODTs) dissolve rapidly in the mouth, aiding elderly patients and those with dysphagia. Cocoa powder effectively masks the bitterness of active ingredients such as rebamipide in ODTs, enhancing palatability, particularly with 2.5 and 10% formulations, as well as improving their clinical palatability (48). Using cocoa butter as a matrix, a study successfully formulated fast melt tablets (FMTs), showcasing rapid disintegration (32.67 s) and excellent drug release (98.07% within 10 min). Stability studies confirmed formulation robustness for 6 months, emphasizing cocoa butter’s potential as an effective matrix material (49), while another study developed FMTs using cocoa butter and caffeine. The optimized formulation disintegrated in 1.20 min, met pharmacopeial standards, and exceeded US FDA drug release requirements, showcasing the refrigerator freezing method’s efficiency (50). A fast-dispersing cocoa-based tablet was developed using freeze-casting, achieving needle-columnar and planar-lamellar pore structures. The addition of sugar enhanced properties, resulting in <1 min dispersal time and > 34.5 N crushing force. The tablet meets dissolution and mechanical strength requirements, highlighting practical potential (51).

In the current research, we developed a novel approach for formulating nutraceutical tablets using a hydroalcoholic extract of defatted cocoa powder through cold maceration. Employing the Quality by Design (QbD) approach and utilizing Design of Experiments (DoE) software ensured a rigorous development process. The critical quality attributes (CQAs) defined for the tablets included loss due to friability below 1.0% and a disintegration time of no more than 15 min. Using a face-centered central composite design, statistical analysis optimized the formulation, reducing risks and ensuring the product met QTPP and CQA standards. The Face-Centered Central Composite Design (FCCCD) is a powerful and popular experimental design technique used in the formulation and optimization of nutraceutical tablets. Within the larger framework of response surface methodology (RSM), the FCCCD facilitates a systematic exploration of the relationships between various formulation and process variables to fine-tune product characteristics. For oral solid dosage forms, the FCCCD is especially beneficial in refining key quality attributes such as hardness, disintegration time, dissolution rate, and stability. The FCCCD effectively explores multiple factors, such as excipient concentrations and compression force, to optimize tablet quality through factorial, axial, and center points. The FCCCD optimizes excipient formulations, such as binders and disintegrants, and adjusts compression force to impact tablet characteristics such as hardness, disintegration time, and dissolution rate (52).

This study focused on the formulation of nutraceutical tablets using the direct compression method, a widely used technique in tablet manufacturing due to its faster production time and ability to preserve the bioactivity of sensitive ingredients such as spray-dried cocoa extract. Preliminary risk analysis identified Avicel PH-101 and croscarmellose sodium concentrations are potential independent variables for selected dependent variables and CQAs. Avicel concentration significantly influences friability by affecting hardness and mechanical strength (53). Higher levels increase hardness, slowing disintegration, while lower levels reduce hardness, speeding it up. Proper Avicel balance ensures optimal dissolution and stability (54). Croscarmellose sodium (CCS) is a crucial super disintegrant that enhances tablet disintegration and bioavailability. However, higher CCS concentrations may increase disintegration time, weakening tablet structure. Adjusting compression force improves hardness, reducing breakage risks (55, 56). Optimizing CCS concentration and compression balance ensures effective disintegration and tablet integrity, highlighting the importance of careful formulation and process optimization. Avicel PH-101’s concentration was found to significantly impact tablet hardness and % loss due to friability, balancing dissolution and stability. CCS, acting as a super disintegrant, accelerated disintegration. Multiple regression and analysis of variance (ANOVA) identified the optimal formulation F9, which met QTPP and CQA standards, comprising croscarmellose sodium (5%) and Avicel PH-101 (35%). F9 had a disintegration time of 5.2 min and % loss due to friability of 0.34%, which was developed again and evaluated for its responses revealing a disintegration time of 5.1 min and % loss due to friability of 0.35%. Toxicity tests following OECD Test Guideline 423 confirmed that it is safe for oral administration. The novelty of the developed formulation lies in the strategic use of Avicel PH-101 and CCS to achieve an optimal balance between disintegration time and friability. This approach, guided by QbD principles and insights from DoE analysis, highlights the potential for a reliable and convenient dosage form. Our research advances nutraceutical tablet formulations and showcases the versatility and potential of cocoa-based products in the nutraceutical field.

In addition, the measurement of (+)-catechin (CTN) and (−)-epigallocatechin-3-gallate (EGCG) in the quality control of nutraceutical tablets or any other formulations or preparation containing these bioactive compounds can be done using the developed novel simultaneous CTN and EGCG HP-TLC method. HP-TLC is an efficient chromatographic method used for both qualitative and quantitative analyses, commonly applied in industries such as pharmaceuticals, food, and environmental testing. Compared to other techniques such as high-performance liquid chromatography (HPLC), gas chromatography (GC), and UHPLC–MS, HP-TLC stands out due to its affordability, requiring less solvent and simpler equipment. It also offers quicker analysis, is easier to use, and consumes smaller sample amounts. In addition, HP-TLC is non-destructive, allowing for further testing of the sample, and provides visual results that are sufficient for many applications (57, 58).

A study developed a validated simultaneous HP-TLC method for quantifying epigallocatechin-3-gallate (EGCG) and rosmarinic acid (RA), employing silica gel-coated plates and a mobile phase of ethyl acetate, toluene, formic acid, and methanol (4:4:1:1 v/v), and the method achieved precise separation (Rf = 0.38 for EGCG) (59); in another study, HP-TLC analysis revealed significant antioxidant potential in the hydroalcoholic extract of *Acacia suma*, using silica gel plates (60 F-254) and a mobile phase of THF/toluene/acetic acid/water [16:8:2:1 (v/v)], and epigallocatechin was identified with an Rf value of 0.944 at 269 nm (60). However, HP-TLC fingerprints for green tea samples were developed using the mobile phase toluene:acetone:formic acid (9:9:2, v/v/v), and EGCG was identified with an Rf value of 0.35 after derivatization with Fast Blue Salt B reagent (61). An HP-TLC analysis of *Parkia roxburghii* seed extract quantified catechin using a solvent system of ethyl acetate, acetic acid, formic acid, and water (10:1:0.75:1, v/v). Catechin was observed at an Rf value of 0.61, with a content of 2.24% w/w (62). Our novel HP-TLC method achieved good separation of CTN and EGCG, with Rf values of 0.29 and 0.45, respectively. The improved band separation makes it suitable for standardizing alternative herbal extracts containing these bioactive compounds.

Our chromatographic analysis aimed to separate CTN and EGCG using HP-TLC. The most effective mobile phase was ethyl ethyl acetate/toluene/formic acid (9:9:2 % v/v), providing a clear resolution of the compounds. The selected mobile phase facilitated the optimal migration of catechins on the TLC plate. Precise sample application and controlled plate development ensured reproducibility and accuracy. Densitometric analysis revealed Rf values of 0.45 for (+)-catechin and 0.29 for (−)-epigallocatechin, indicating differences in polarity and interactions with the stationary phase. The results demonstrate that the ethyl acetate/toluene/formic acid (9:9:2 % v/v) mixture effectively separates catechins, validating the method’s reliability for qualitative or quantitative analysis. After separation, the spots corresponding to (+)-catechin and (−)-EGCG are visualized under UV light at 254 nm as both compounds absorb UV light. CTN typically forms a distinct spot, while EGCG displays a clear UV–visible band. The TLC plate is then scanned using a densitometer (CAMAG TLC Scanner 3), which measures the intensity of the spots. The plate is then positioned in the CAMAG TLC Scanner 3, which is equipped with UV light at 254 and 366 nm, R-white light, and both deuterium and tungsten lamp detectors to measure the intensity of the spots. The TLC Scanner 3 scans the plate from one end to the other, detecting the light reflected from each spot. It follows a predetermined scanning pattern, typically using a fixed wavelength of 254 nm for detection. The densitometer records the reflected light intensity, which correlates with the concentration of the compound in each spot. This information is then converted into a densitogram, a graphical display of the peaks representing each compound. The amounts of CTN and EGCG are quantified based on the densitometric analysis of the spot areas on the HP-TLC plate. The quantification results of 22.43 μg for CTN and 18.20 μg for EGCG are vital for quality control, confirming that the therapeutic components are present at the appropriate concentrations in the final product. Overall, the HP-TLC results confirm the method’s efficacy in providing detailed profiles of bioactive compounds, facilitating consistent quality in nutraceutical products. This novel approach not only enhances the reliability of herbal product formulations but also sets a new standard for the quality control of nutraceuticals, thus demonstrating HP-TLC method developed was effective, reproducible, and specific for (+)-catechin and (−)-epigallocatechin-3-gallate for validating and quantifying the polyphenol content in both the extract and formulation prepared.

## 5 Conclusion

In our previous studies, CE demonstrated a significant reduction in the toxicity induced by cancer chemotherapeutic drugs such as doxorubicin and exhibited anticancer activity in various rodent models. In the current study, we developed and optimized an orally administered formulation as nutraceutical supplement using the QbD approach, with predefined QTPP and CQAs. The optimized formulation, determined through central composite design (CCD), resulted in a disintegration time of 5.2 min and loss due friability of 0.34%. The tablets had a thickness of 4.10 ± 0.08 mm, hardness of 4.7 ± 0.054 kg/cm^2^, and an average weight of 252 ± 1.4 mg, with no observable oral toxicity. We validated a marker-based HP-TLC method using (+)-catechin (CTN) and (−)-epigallocatechin-3-gallate (EGCG), quantifying 22.43 μg (CTN) and 18.20 μg (EGCG) with Rf values of 0.45 (CTN) and 0.29 (EGCG). This method enabled quantification and standardization of these selected polyphenols present in the *Theobroma cacao* L. hydroalcoholic extract and in the developed formulation. However, further long-term stability studies are recommended for the development of commercially viable, long-term storage tablets. Subsequent clinical studies are necessary to explore the use of CE formulation as nutraceutical in palliative therapy in cancer patients.

## Data Availability

The raw data supporting the findings of this study will be made available by the corresponding author upon reasonable request, without undue restriction.
