# Supplementary figures and images for: Quality by design-based optimization and HP-TLC densitometric standardization of Theobroma cacao L. extract as a nutraceutical supplement
Source: Front Nutr. 2025 Apr 9;12:1537963. doi: 10.3389/fnut.2025.1537963 (PMC12016216; doi:10.3389/fnut.2025.1537963)

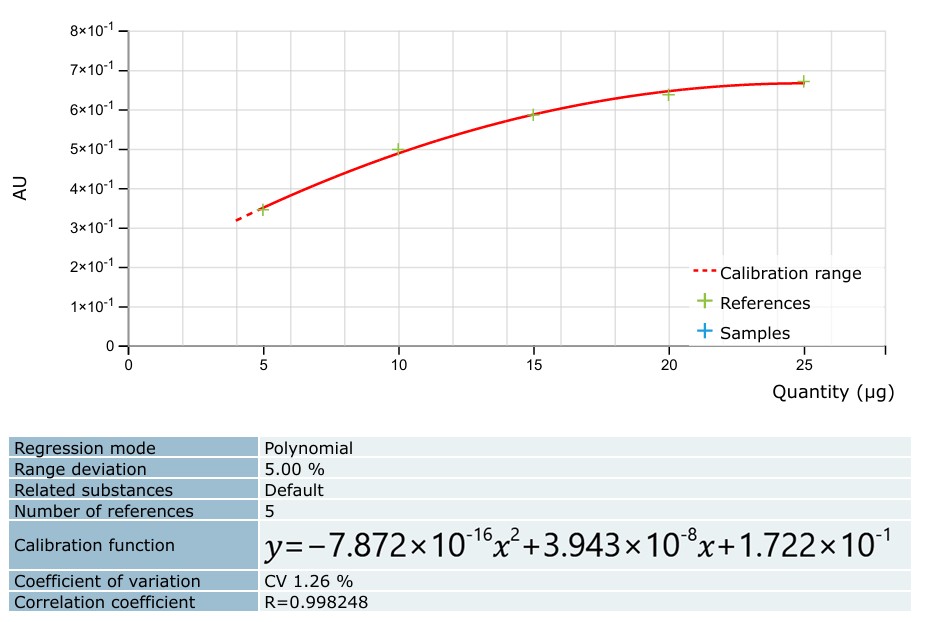

Supplement: Supplementary file 1 [file Image_1.jpeg]

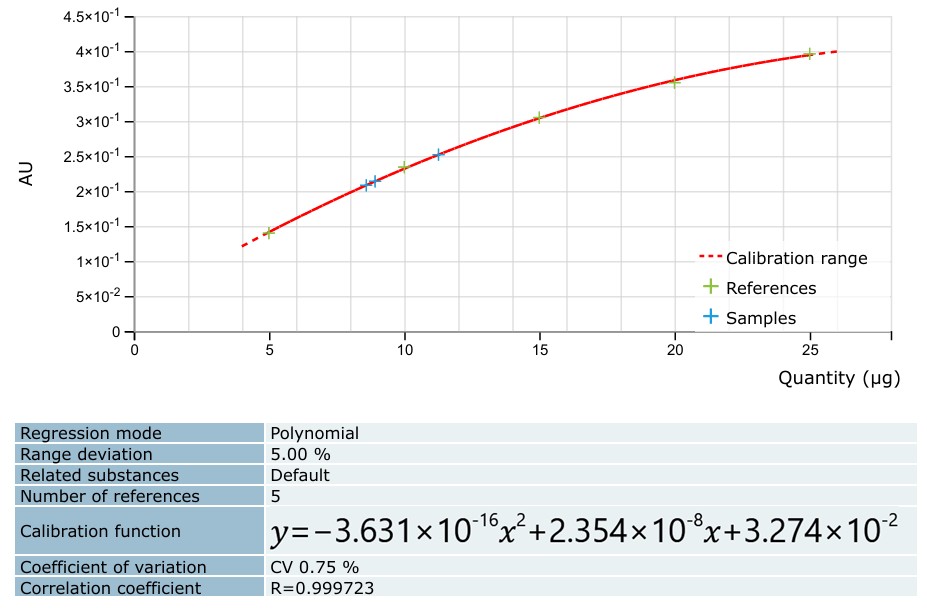

Supplement: Supplementary file 2 [file Image_2.jpeg]
